# Anti-osteoporotic Effect of *Solanum xanthocarpum* Leaf Extract on Retinoic Acid-induced Osteoporosis in Rats via Alleviating Osteoclastic Activity

**DOI:** 10.5812/ijpr-169247

**Published:** 2026-08-26

**Authors:** Shuaishuai Wei, Jiayin Wang, Yi Liu, Chuang Xu, Juncheng Zheng, Periyannan Velu, Annamalai Vijayalakshmi, Dapeng Han

**Affiliations:** 1 Department of Minimally Invasive Spinal Surgery, Public Health Clinical Center Affiliated to Shandong University Jinan China; 2 Department of outpatient, Shandong Armed Police Corps Hospital Jinan China; 3 Department of Research and Development, Vinayaga Research Centre Tamilnadu India

**Keywords:** Osteoporosis, Solanum Xanthocarpum, Retinoic Acid, Osteoblast, Osteoclast, NOX1/NOX2, SOD1/SOD2, Bone Mineral Density

## Abstract

**Background:**

Osteoporosis (OP) is a metabolic bone disorder that predominantly affects postmenopausal women. *Solanum xanthocarpum* (SX), a wild herb in the Solanaceae family, has traditionally been used to manage postmenopausal symptoms and articular cartilage injury.

**Objectives:**

This study aimed to evaluate the anti-osteoporotic activity of *Solanum xanthocarpum* leaf extract (SXLE) in a rat model of retinoic acid (RA)-induced osteoporosis.

**Methods:**

Female Sprague-Dawley (SD) rats were randomly assigned to six experimental groups (n = 6 per group): group I, control; group II, RA alone (75 mg/kg body weight/day); group III, RA + alendronate (5 mg/kg body weight); group IV, RA + SXLE (25 mg/kg body weight); group V, RA + SXLE (50 mg/kg body weight); and group VI, RA + SXLE (100 mg/kg body weight). Bone mineral density (BMD), bone length, bone mineral content, serum estradiol levels, osteocalcin (OCN), alkaline phosphatase (ALP), and tartrate-resistant acid phosphatase (TRAP) activity were measured. The mRNA expression levels of TNF-α, IL-6, TP53 (tumor protein p53), NOX1 (NADPH oxidase 1), NOX2 (NADPH oxidase 2), SOD1 (superoxide dismutase 1), and SOD2 (superoxide dismutase 2) were evaluated by RT-qPCR.

**Results:**

SXLE treatment significantly increased body weight, bone mineral content, bone length, relative bone density, serum estradiol, osteocalcin, and BMD in a dose-dependent manner. Conversely, SXLE dose-dependently reduced ALP and TRAP activities and decreased the mRNA expression of TNF-α, IL-6, and TP53. SXLE improved tibial and femoral coefficients and lengths, increased trabecular relative bone density, and enhanced the cortical bone area ratio. The therapeutic effects of SXLE at 100 mg/kg body weight were comparable to those of alendronate (5 mg/kg body weight). Furthermore, SXLE downregulated the expression of NOX1 and NOX2 while upregulating the expression of SOD1 and SOD2.

**Conclusions:**

SXLE enhances osteoblastic/osteogenic activity while suppressing osteoclastic activity, indicating significant anti-osteoporotic potential in rats with RA-induced osteoporosis. This is the first report to link SXLE to modulation of the NOX1/NOX2-SOD1/SOD2 oxidative stress pathway in bone, providing a potential molecular mechanism for its bone-protective effects. In addition, SXLE at 100 mg/kg demonstrated therapeutic efficacy comparable to that of alendronate, supporting its potential as a plant-based alternative to bisphosphonates for osteoporosis management.

## 1. Background

Osteoporosis (OP) is a progressive bone disorder characterized by excessive bone resorption, low bone mass and bone mineral density (BMD), and deterioration of trabecular microarchitecture, which increase bone fragility and susceptibility to fracture (1, 2). Approximately 1.24 million fractures caused by OP are reported globally each year (3), and the disorder predominantly affects older adults (3, 4). In Asia, particularly in China, the prevalence of OP is increasing steadily (3). Women are at greater risk after menopause because of accelerated bone turnover (5, 6). Although the etiology of OP is multifactorial, estrogen plays a central role in bone homeostasis and skeletal development through direct and regulatory effects on bone cells. Consequently, estrogen deficiency at menopause reduces osteoprotective effects and contributes to postmenopausal OP (7). The primary goal of OP treatment is to prevent fractures by reducing bone loss and improving bone strength and density. Early recognition and treatment can reduce the risk of subsequent bone disorders, although restoring lost bone remains difficult (8, 9). Therefore, timely prevention and treatment of OP are important for limiting bone damage (9).

The development of OP involves multiple factors, including inflammation and oxidative stress, that disrupt bone remodeling by increasing resorption (10, 11). Inflammatory cytokines, particularly tumor necrosis factor-alpha (TNF-α), interleukin-6 (IL-6), and interleukin-1 beta (IL-1β), play important roles in OP. TNF-α promotes receptor activator of nuclear factor kappa-B ligand (RANKL) signaling, thereby enhancing osteoclast differentiation and bone resorption (12). Increased osteoclast activity and resorption are key pathological features of OP. Bone undergoes continuous remodeling through coordinated osteoblast and osteoclast activity (13, 14); an imbalance favoring osteoclast-mediated resorption over osteoblast-mediated formation results in bone loss (14). Bone-remodeling biomarkers include enzymes or products released by osteoblasts and osteoclasts during their development and are commonly measured in plasma or serum to monitor responses to osteoanabolic or antiresorptive agents (15). Thus, bioactive compounds with antioxidant and anti-inflammatory activities may be useful in OP management.

Plant-based natural bioactive compounds containing polyphenols and flavonoids have been shown to exert anti-OP effects, preventing bone loss in various rat models (16, 17). Phenolic compounds are important secondary plant metabolites and exhibit potent anti-inflammatory (18), antioxidant (19), and hepatoprotective (20) properties. Additionally, numerous reports have shown that phenolic extracts from diverse plants, including grapes (21), olives (22), black beans (23), and tea (24), exhibit anti-OP activities. *Solanum xanthocarpum* (SX) is a wild herb belonging to the family Solanaceae that is cultivated in several provinces of India. In Ayurvedic preparations, different parts of SX have been used, including root decoction, stem, fruit extracts (FE), and leaf extracts (LE), for diverse therapeutic effects (25). *Solanum xanthocarpum* leaf extracts (SXLE) have been investigated for larvicidal, antimicrobial, and wound-healing activities (26). The SX plant contains several active constituents, including steroidal alkaloids such as solanacarpidine, solamargine, solanacarpine, solasonine, and solancarpine, as well as coumarins (aesculin and aesculetin), caffeic acid, steroids (diosgenin, carpesterol, daucosterol, and campesterol), and triterpenes (cycloartanol and cycloartenol) (27). The pharmacological activities of SX, such as hypotensive, hypoglycemic (28), and hepatoprotective (26) effects, have been examined. As an antioxidant, SXLE shows in vitro anticancer and anti-obesity activities. Earlier reports also described the antioxidant and anti-inflammatory potential of SX seeds and leaves (29). Another study reported that SX fruit extract ameliorated osteoarthritis by promoting chondrocyte proliferation and preventing articular cartilage injury through suppression of COX-2 and MMP-3 expression (30). A previous study also suggested that an aqueous SX extract may help alleviate postmenopausal symptoms in bilaterally ovariectomized rats (31). These findings support phenolic compounds as important anti-inflammatory constituents of SX and provide a scientific basis for further investigation of its bioactive components (32). However, the anti-osteoporotic activity of the phenolic fraction of SXLE remains largely unknown.

Retinoic acid (RA), a derivative of vitamin A, is used for skin disorders and tumors and also participates in skeletal and hematopoietic development (33). RA-induced OP in rats is widely used to evaluate the effects of candidate compounds on bone injury (33, 34). High vitamin A exposure is detrimental to the skeleton in animal models, and RA can cause a sustained reduction in BMD over 1 - 3 weeks (35). The RA-induced model is frequently used because it is simple, rapid, inexpensive, reproducible, and produces OP-like histomorphological changes (34, 35). NADPH oxidase 1 (NOX1) and NADPH oxidase 2 (NOX2) contribute to reactive oxygen species (ROS) generation in RA-induced OP. In the presence of RA, the pro-osteoclastogenic cytokine RANKL activates NOX1 and NOX2 to generate superoxide, promoting osteoclast activity and suppressing osteoblast activity (36). However, the role of NOX signaling in the anti-osteoporotic effects of the phenolic extract of SXLE has not been established.

## 2. Objectives

In the present study, we investigated the antioxidant and anti-inflammatory effects of the SXLE phenolic extract in RA-induced OP in rats, with a focus on the regulation of the NOX1/NOX2 and superoxide dismutase 1 (SOD1)/superoxide dismutase 2 (SOD2) pathways. This study provides additional evidence supporting the potential use of the SXLE phenolic extract in the management of bone-related disorders and its effects on bone-remodeling markers.

## 3. Methods

### 3.1. Chemicals

Chloral hydrate, phosphate-buffered saline (PBS), and retinoic acid (RA) were obtained from BioReal (Sweden). Alendronate was purchased from Novartis Pharmaceuticals (NJ, USA). Biochemical assay kits were supplied by Solarbio (Beijing, China), and other biochemicals and reagents were obtained from ZSGB-Bio (Beijing, China). *Solanum xanthocarpum* leaves (SXL) were collected in India, and the plant was identified by a botanist.

### 3.2. Preparation of *Solanum Xanthocarpum* Leaf Extracts

*Solanum xanthocarpum* leaves were collected and transported to the laboratory. The leaves were thoroughly washed under running water to remove soil and other foreign materials, shade-dried, and powdered. A total of 100 mg of dried leaf powder was sequentially extracted with hexane, ethyl acetate, and methanol using a Soxhlet apparatus (DWK Life Sciences, NJ, USA). The crude extract was concentrated to dryness using a flash evaporator. The methanolic extract of *S. xanthocarpum* leaves (SXLE-MeOH) was used for all in vivo experiments. The dried extracts were stored in a refrigerator for subsequent experiments (37).

### 3.3. Determination of Total Phenolic Content

The total phenolic (TP) content of the SXLE preparations was estimated using the Folin-Ciocalteu reagent as previously described (38). Each extract (50 mL) was treated with 2.5 mL of a 1:10 dilution of Folin-Ciocalteu reagent and 2 mL of 7.5% Na_2_CO_3_ and mixed thoroughly. The mixture was incubated at 45 °C for 15 min, and absorbance was measured at 765 nm using Na_2_CO_3_ solution as the blank. Results were expressed as gallic acid equivalents (GAE). The TP content of SXLE-MeOH was 125.4 ± 3.2 mg GAE/g.

### 3.4. Experimental Animals

Adult female Sprague-Dawley (SD) rats weighing 200 - 220 g were used. The animals were housed in disinfected polypropylene cages under controlled conditions, provided a standard pellet diet ad libitum, and maintained on a 12-hour light/dark cycle. Rats were acclimatized for 1 week before the experiments. All treatment protocols were conducted in accordance with institutional animal ethics guidelines.

### 3.5. Experimental Plan

OP was induced according to a previously described method (39). Rats were randomly assigned to 6 groups of 6 animals each. Group I served as the normal control (NC). Group II received RA alone (75 mg/kg body weight/day) via intragastric administration for 2 weeks. Group III received RA + alendronate (AL, 5 mg/kg body weight) orally each day. Adult female Sprague-Dawley rats in groups IV-VI received RA + SXLE-MeOH at 25, 50, or 100 mg/kg body weight, respectively. Individual body weight was recorded to adjust the oral dose. At the end of the experiment, animals were fasted overnight, anesthetized with chloral hydrate, and blood was collected. Serum was separated by centrifugation at 4 °C for 5 min at 2000 r/min.

### 3.6. Biochemical Assays

Serum and urinary phosphorus (P) and calcium (Ca), as well as serum alkaline phosphatase (ALP), were measured using commercial kits from Solarbio (Beijing, China) according to the manufacturer's protocols.

### 3.7. Measurement of Organ Coefficients

Organ coefficients were determined as previously described (39). The femur, tibia, and liver were isolated from each rat and weighed. Adherent tissues, including tendons, muscle, and fat, were removed with a sterile surgical blade while retaining the bone marrow. The left femur and tibia were then dried for 2 hours at 110 °C and weighed. Organ coefficients were calculated as follows: Organ coefficient = Organ Wt /Body Wt ×100.

### 3.8. Analysis of Bone Features

Bone-quality indices were evaluated using the right femur and tibia. The assessed parameters included bone length, bone calcium content, BMD, and maximum load. Femur and tibia lengths were measured using an electronic vernier caliper (SKADIOO Electronic Digital Caliper). Whole-bone BMD was measured using a microcomputed tomography scanner (PerkinElmer, USA). Bone calcium content was estimated by flame atomic absorption spectrometry (PerkinElmer, USA). Maximum load was measured using a universal testing machine (Zhejiang Tugong Instrument Co., Ltd., China) with a three-point bending test. The cortical bone area ratio and trabecular relative bone density were determined using a previously reported method (39).

### 3.9. Evaluation of Biochemical Factors

Serum estradiol, osteocalcin (OCN), and TRAP were assessed using specific commercial kits (Solarbio, Beijing, China) according to the manufacturer's instructions.

### 3.10. Histopathological Study

The right femur, liver, and kidney tissues were rapidly removed and fixed in 10% buffered formalin for 1 day. Bone tissue was decalcified, and tissues were subsequently paraffin-embedded. Sections were cut and stained with hematoxylin and eosin (H&E) for microscopic examination. An experienced pathologist performed the histological evaluation of all experimental groups.

### 3.11. Masson's Trichrome Staining

Masson's trichrome staining of femur/tibia tissue was used to differentiate collagen (blue), muscle (red), and nuclei (black). Deparaffinized sections were stained with Weigert's iron hematoxylin for 10 min, Biebrich scarlet-acid fuchsin for 5 - 10 min, differentiated in phosphomolybdic acid for 10 min, stained with aniline blue for 5 min, fixed in 1% acetic acid, dehydrated, and mounted.

### 3.12. RT-qPCR Analysis of mRNA Expression

Total RNA was isolated from femur/tibia bone tissue using the TRIzol® kit (Promega, Madison, WI, USA) according to the manufacturer's instructions. Equal amounts of RNA were used for cDNA synthesis with a cDNA synthesis kit (Solarbio, Beijing, China) (Table 1). RT-qPCR was performed using SYBR Green Supermix (Abcam, USA). Gene expression was determined using the 2^−ΔΔCT^ method and normalized to the internal control GAPDH. Primer sequences were as described previously (36).

**Table 1.** Primer Sequences Used in Real-Time PCR Assays

Genes	Forward sequences	Reverse sequences
**IL-6**	5’-AGACAGCCACTCACCTCTTCAG-3’	5’-TTCTGCCAGTGCCTCTTTGCTG-3’
**TNF-α**	5’-CTCTTCTGCCTGCTGCACTTTG-3’	5’-ATGGGCTACAGGCTTGTCACTC-3’
**TP53**	5’-CCTCAGCATCTTATCCGAGTGG-3’	5’-TGGATGGTGGTACAGTCAGAGC-3’
**NOX-1**	5’-GGTTTTACCGCTCCCAGCAGAA-3’	5’-CTTCCATGCTGAAGCCACGCTT-3’
**NOX-2**	5’-ATGGAGGTGGGACAATACA-3’	5’- CAGACTTGAGAATGGAGGC-3’
**SOD1**	5’-CTCACTCTCAGGAGACCATTGC-3’	5’-CCACAAGCCAAACGACTTCCAG-3’
**SOD2**	5’-CTGGACAAACCTCAGCCCTAAC-3’	5’-AACCTGAGCCTTGGACACCAAC-3’
**GAPDH**	5’-GAC GGC CGC ATC TTC TTG T-3’	5’-CAC ACCGACCTT CAC CATTTT-3’

### 3.13. Statistical Analysis

Data were analyzed using GraphPad Prism version 9.0. One-way analysis of variance followed by Duncan's multiple range test was used for comparisons among multiple groups, whereas Student's t-test was used for comparisons between 2 groups. A P < 0.05 was considered statistically significant. Results are expressed as the mean ± standard deviation (SD) from n = 6 independent experiments.

## 4. Results

### 4.1. Effects of SXLE Treatments on Weekly Body Weight

At baseline (week 0), mean body weights ranged from 205 to 216 g across groups and increased over 4 weeks in all groups (Figure 1). RA induction significantly suppressed weight gain compared with the control group at all time points (control, 230 g vs RA, 217 g at week 4). AL (5 mg/kg body weight) largely restored weight gain (RA + AL, 224 g at week 4). SXLE produced a dose-dependent recovery; 25, 50, and 100 mg/kg resulted in progressive improvements, with the 100 mg/kg group reaching 223 g at week 4.

**Figure 1.**
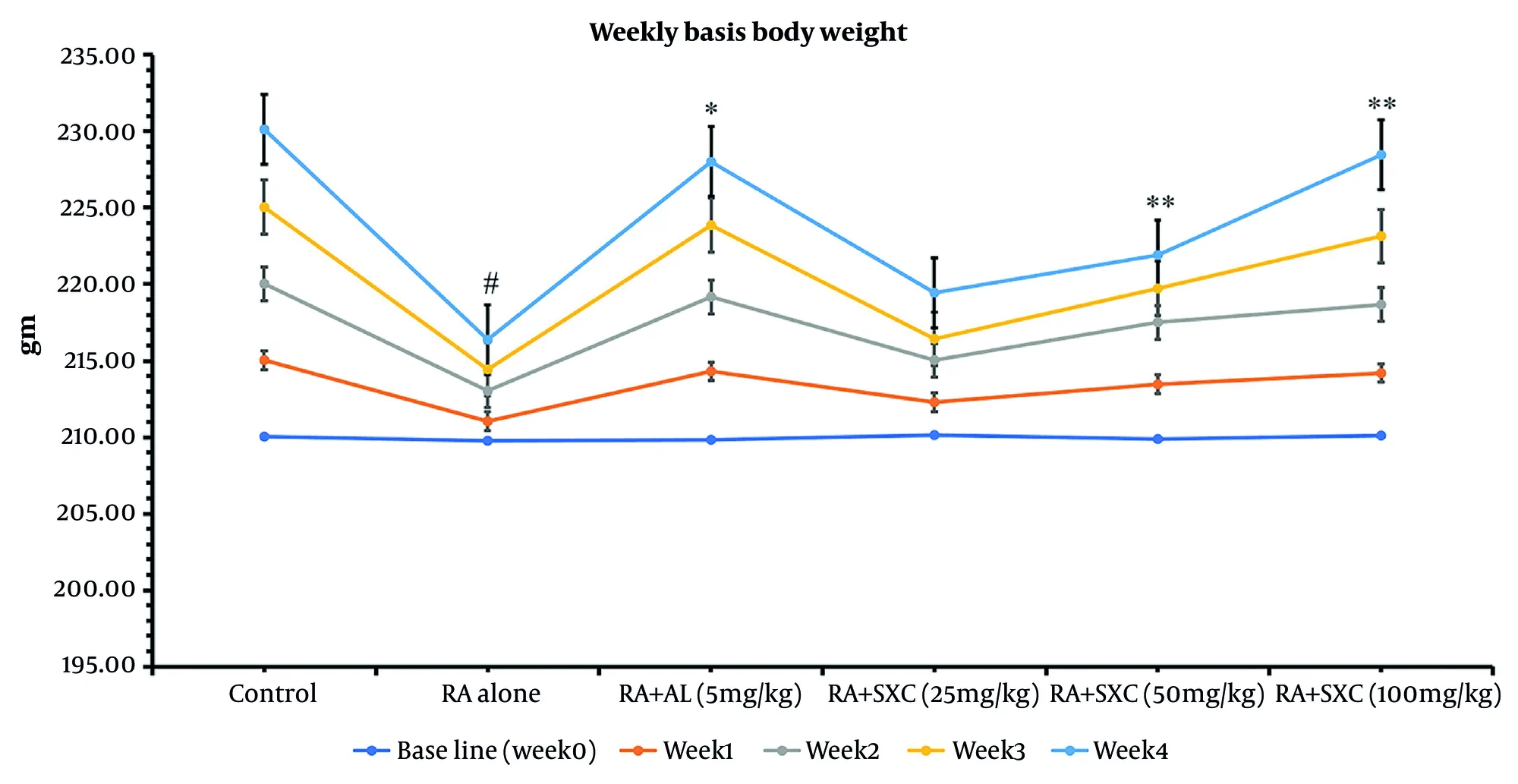
Weekly body-weight changes over 4 weeks. Control, RA, RA + AL (5 mg/kg bw), and RA + SXLE (25, 50, and 100 mg/kg bw) groups from baseline (week 0) to week 4. Data are presented as mean ± SD of 6 observations. Statistical significance is indicated as # P < 0.05 vs NC, * P < 0.05 vs RA alone, and ** P < 0.01 vs RA alone.

### 4.2. Effects of SXLE on Bone Minerals and Marker Enzymes

Compared with the NC group, the RA-induced group showed significantly increased serum calcium, phosphorus levels (P < 0.05), urinary phosphorus and calcium levels (P < 0.05) (Figure 2A-D). Administration of SXLE (25, 50, and 100 mg/kg body weight) to RA-treated rats dose-dependently lower serum calcium and phosphorus levels (P < 0.05) and reduced urinary calcium and phosphorus levels (P < 0.05) compared with the RA-alone group.

**Figure 2.**
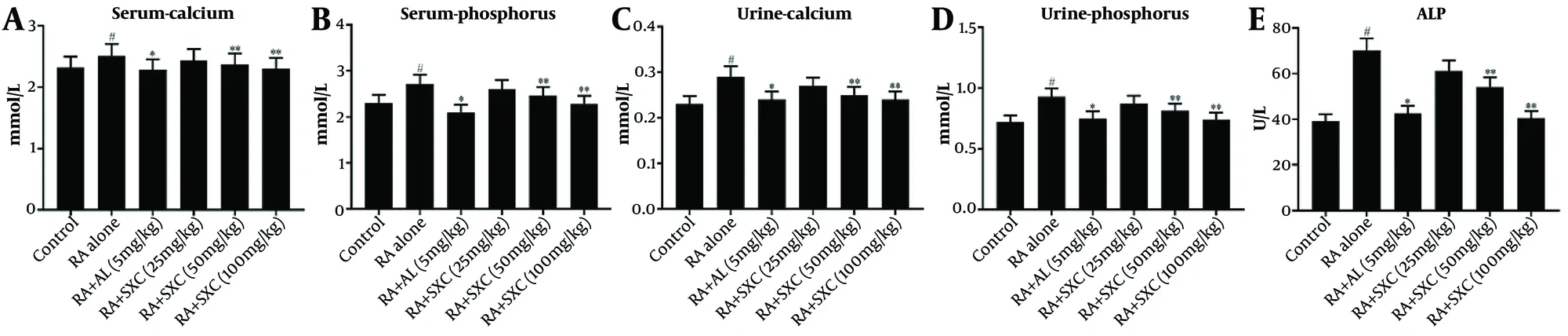
Protective effect of SXLE on RA-induced OP rats; A, Serum calcium; B, serum phosphorus; C, urine calcium; D, urine phosphorus; E, ALP. Data are presented as mean ± SD of 6 observations, and statistical significance is indicated as # P < 0.05 vs NC, * P < 0.05 vs RA alone, and ** P < 0.01 vs RA alone

ALP activity was significantly elevated (P < 0.05) in the RA-induced group compared with the NC group (Figure 2E). Treatment with SXLE (25, 50, and 100 mg/kg body weight) significantly reduced RA-induced ALP activity (P < 0.05) in a dose-dependent manner .

### 4.3. Effects of SXLE on Organ Coefficients

Retinoic acid (RA) injection dramatically decreased the dry femur (Figure 3A) and tibia (Figure 3B) coefficients when compared to the normal control group, indicating that the RA model was successfully induced. In comparison to the RA-alone group, treatment with alendronate (AL, 5 mg/kg) or SXC (25, 50, or 100 mg/kg) significantly raised both bone coefficients; the 50 and 100 mg/kg SXC doses restored values near control levels. Additionally, the Liver Coefficient. In comparison to controls, RA induction significantly increased the liver coefficient, which is a symptom of hepatomegaly or hepatic stress. This rise was considerably reduced by co-administration of AL (5 mg/kg) or SXC (25, 50, or 100 mg/kg), bringing liver coefficients back to normal control values.

**Figure 3.**
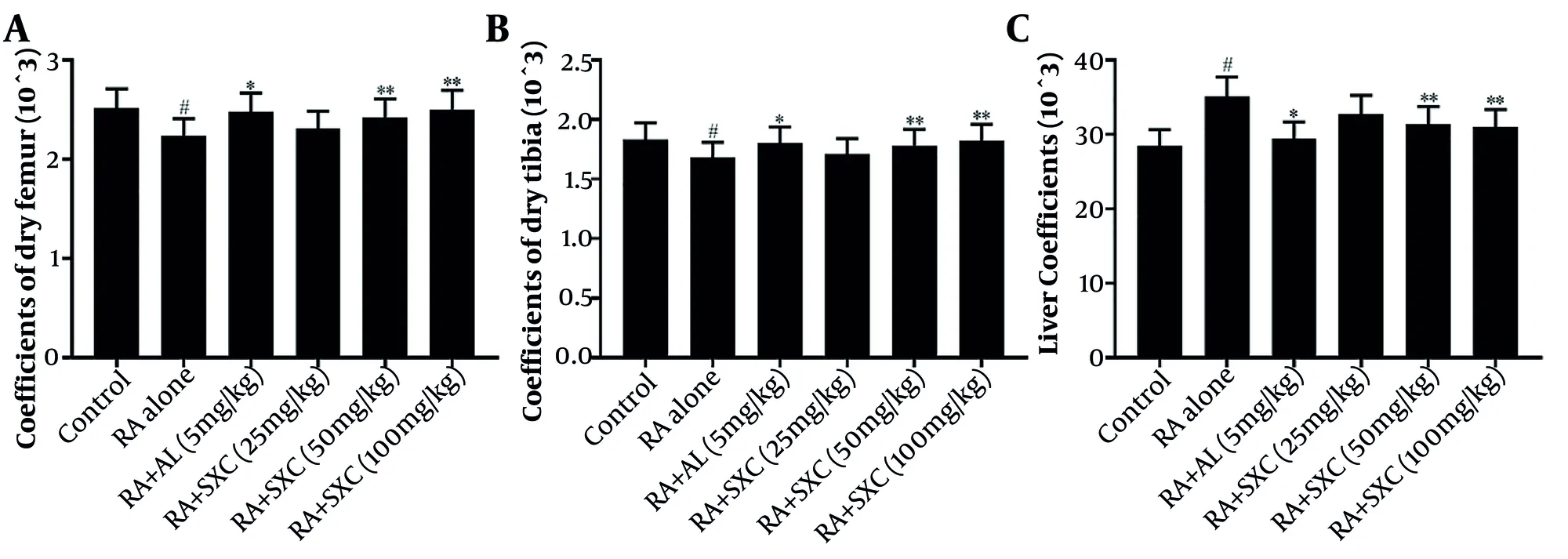
Effect of SXLE on organ coefficients of RA-induced OP rats; A, Coefficient of dry femur; B, coefficient of dry tibia; C, liver coefficient. Data are presented as mean ± SD of 6 observations, and statistical significance is indicated as # P < 0.05 vs NC, * P < 0.05 vs RA alone, and ** P < 0.01 vs RA alone

### 4.4. SXLE Enhances Bone Quality

Morphometric, density, mineral, and biomechanical parameters were examined in femur and tibia tissues to assess the therapeutic effect of SXC on retinoic acid (RA)-induced bone degradation (Figure 4A-H). As demonstrated by significant decreases in femur length (Figure 4A), tibia BMD (Figure 4D), bone calcium content in both femur (Figure 4E) and tibia (Figure 4F), and maximum load capacity in both bones (Figure 4G, Figure 4H), RA administration significantly (#P < 0.05) compromised bone integrity when compared to the Control group. On the other hand, RA challenge resulted in abnormal increases in femur BMD (Figure 4C; # P < 0.05) and tibia length (Figure 4B). When compared to the RA-alone group, SXC co-treatment dose-dependently reduced these pathological alterations. Higher doses of SXC (50 mg/kg,*P < 0.05; 100 mg/kg, * P < 0.05 or P < 0.01) significantly normalized length measurements, restored baseline BMD profiles, restored calcium mineral density, and increased maximum mechanical load capacity in both bones, whereas the low dose (25 mg/kg) showed negligible recovery. Significantly, the therapeutic recovery attained with 100 mg/kg SXC was similar to that of the positive control group (RA + AL, 5 mg/kg; * P < 0.05), indicating that SXC has dose-dependent osteoprotective activity against bone deterioration caused by RA.

**Figure 4.**
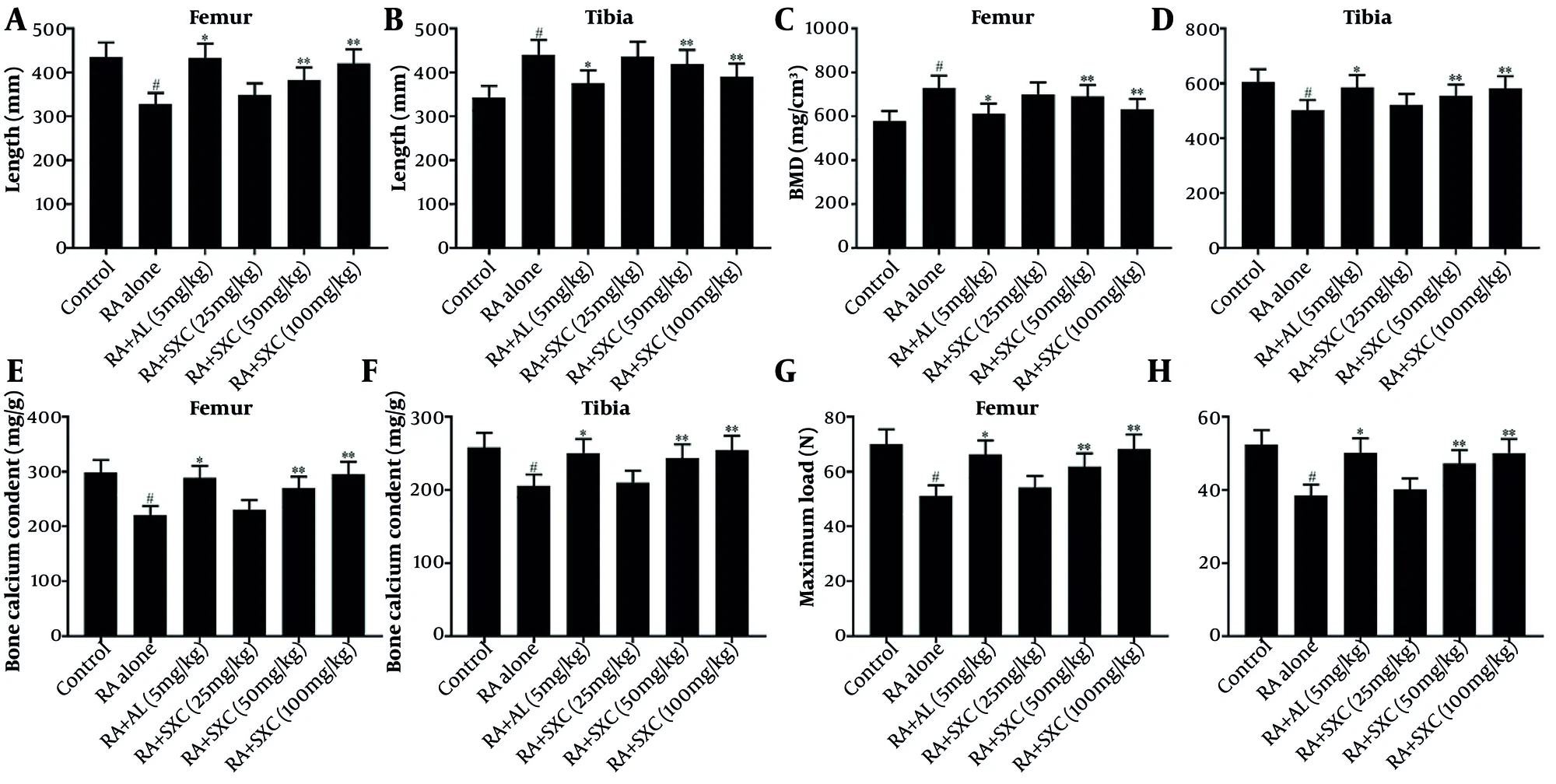
Protective effect of SXLE on bone quality; A, Femur length; B, tibia length; C, femur BMD; D, tibia BMD; E, femur bone calcium content; F, tibia bone calcium content; G, maximum load of femur; H, maximum load of tibia. Data are presented as mean ± SD of 6 observations, and statistical significance is indicated as #P < 0.05 vs NC, *P < 0.05 vs RA alone, and **P < 0.01 vs RA alone

### 4.5. SXLE Enhances Bone-Quality Features

Bone-quality features, such as relative bone density and cortical bone area ratio, were significantly decreased (P < 0.05) in RA-treated rats compared with NC rats (Figure 5A and B). Administration of SXLE (25, 50, and 100 mg/kg body weight) to RA-treated rats dose-dependently increased (P < 0.05) relative bone density and the cortical bone area ratio compared with the RA-alone group. These findings support the osteoprotective efficacy of SXLE against RA-induced OP.

**Figure 5.**
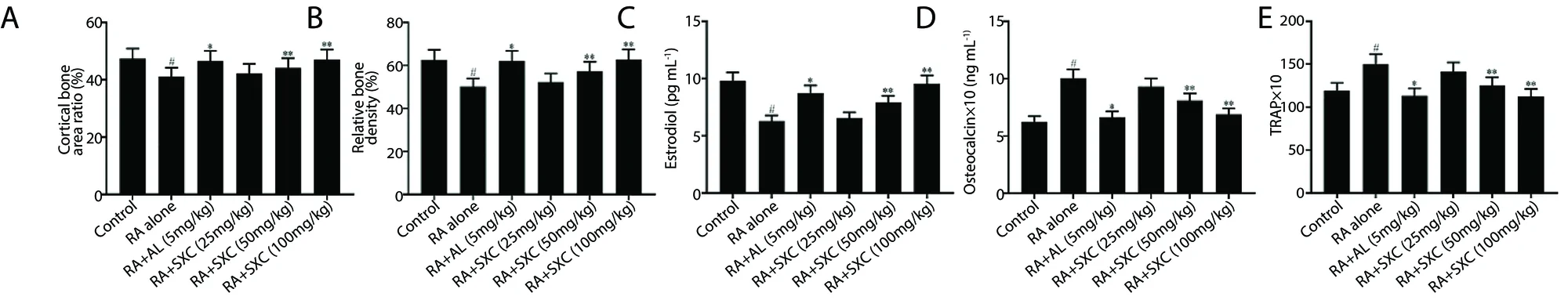
Influence of SXLE on relative bone density and cortical bone area ratio; A, Cortical bone area ratio; B, relative bone density; C, estradiol; D, osteocalcin; E, TRAP. Data are presented as mean ± SD of 6 observations, and statistical significance is indicated as #P < 0.05 vs NC, *P < 0.05 vs RA alone, and ** P < 0.01 vs RA alone

### 4.6. Effects of SXLE on Estradiol, Osteocalcin, and TRAP

Estradiol and osteocalcin levels were significantly decreased (P < 0.05) in RA-induced rats, whereas TRAP activity was increased compared with the NC group (Figure 5C-E). Treatment with SXLE (25, 50, and 100 mg/kg body weight) dose-dependently increased estradiol and osteocalcin (P < 0.05) and decreased TRAP activity (P < 0.05) compared with the RA-induced group. Similar estradiol, osteocalcin, and TRAP values were observed in the RA + AL (5 mg/kg body weight), RA + SXLE (100 mg/kg body weight), and NC groups. These findings indicate that SXLE may suppress RA-induced osteoclastic activity by increasing osteoblastic mediators, such as estradiol and osteocalcin, while reducing the osteoclastic marker TRAP.

### 4.7. SXLE Alleviates RA-Induced Cortical and Trabecular Bone Injury in Rats

H&E staining showed the microarchitecture of trabecular and cortical bone in the right femur of RA-induced rats, including a marked reduction in cortical bone thickness compared with the NC group (Figure 6). Trabecular bone in RA-induced rats was weakened and thinned, with separation and loss of connectivity. SXLE treatment at 25, 50, and 100 mg/kg body weight produced dose-dependent increases in cortical bone thickness, wider trabeculae, a more apparent reticulate structure, and attenuation of abnormal trabecular changes. Treatment with AL (5 mg/kg body weight) or SXLE (100 mg/kg body weight) moderated RA-induced abnormalities in cortical and trabecular bone toward the appearance observed in NC rats. These histopathological findings support the ability of SXLE to attenuate RA-induced bone impairment.

**Figure 6.**
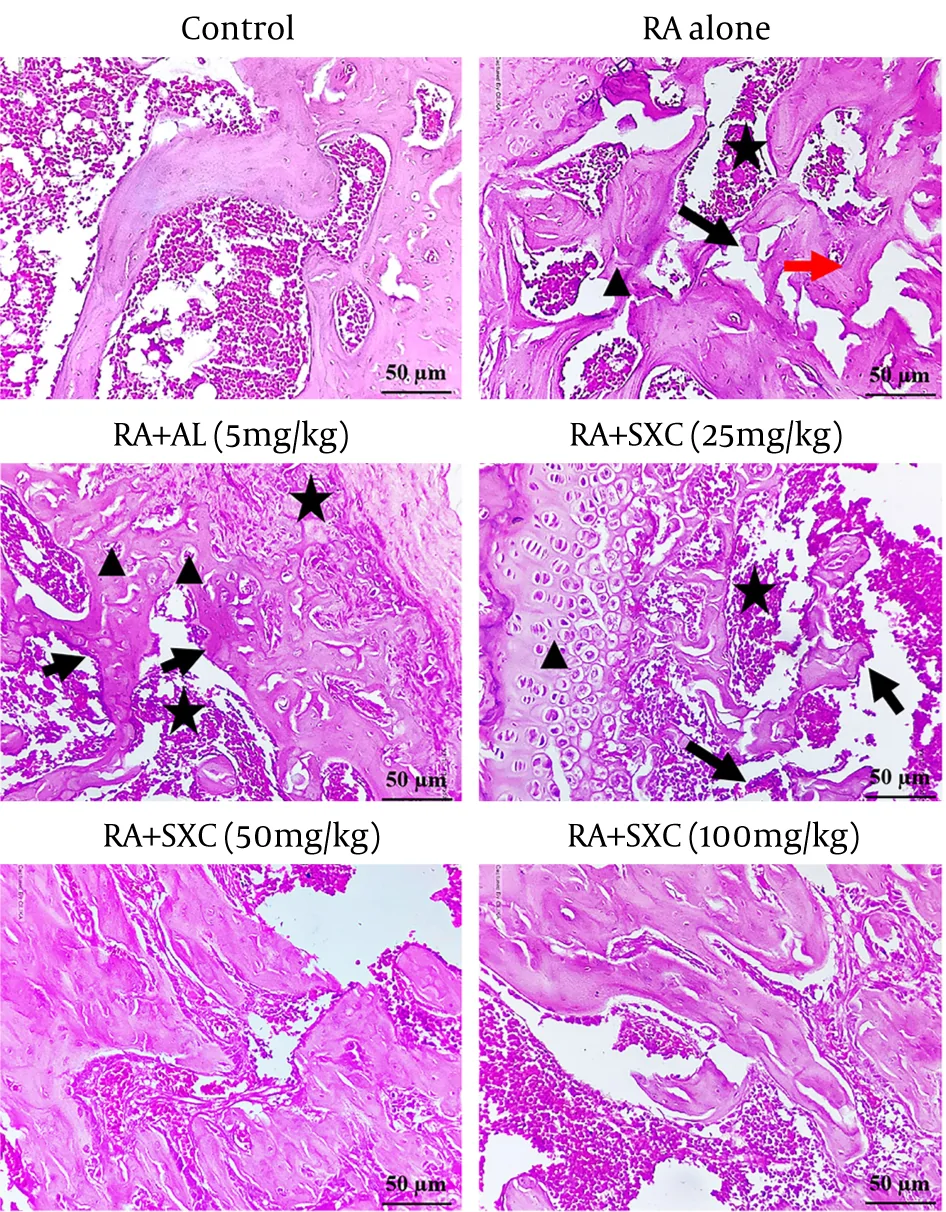
Histopathological assessment of RA-induced bone injury in rats. Representative H&E-stained sections of distal femur (40x, scale bar = 50 μm). NC group: shows normal bone architecture with thick, interconnected trabeculae and intact marrow. RA group: exhibits significant pathological changes including trabecular thinning and fragmentation (▲), inflammatory cell infiltration and marrow congestion (★), and prominent bone resorption pits (black arrows). Severe cortical erosion is also noted (red arrow). SXLE groups: display dose-dependent preservation of bone microarchitecture, evidenced by reduced inflammatory infiltration and improved trabecular continuity compared with the RA group.

### 4.8. Histopathological Assessment of Collagen Deposition (MT Staining)

As shown in Figure 7, RA-induced bone injury was associated with marked disruption of the collagenous matrix compared with the NC group. MT-stained femur sections from the NC group showed well-organized trabecular architecture with dense, continuous blue staining indicative of mature collagen deposition. In contrast, the RA group exhibited pronounced collagen loss, reduced blue staining intensity, fragmented trabeculae, and erosion of the bone matrix in the articular and subchondral regions. SXLE treatment improved these alterations in a dose-dependent manner, with progressively stronger collagen staining and better preservation of trabecular integrity across the treated groups. The highest dose produced the most evident recovery, suggesting a protective effect of SXLE against RA-induced collagen degradation and bone-matrix damage.

**Figure 7.**
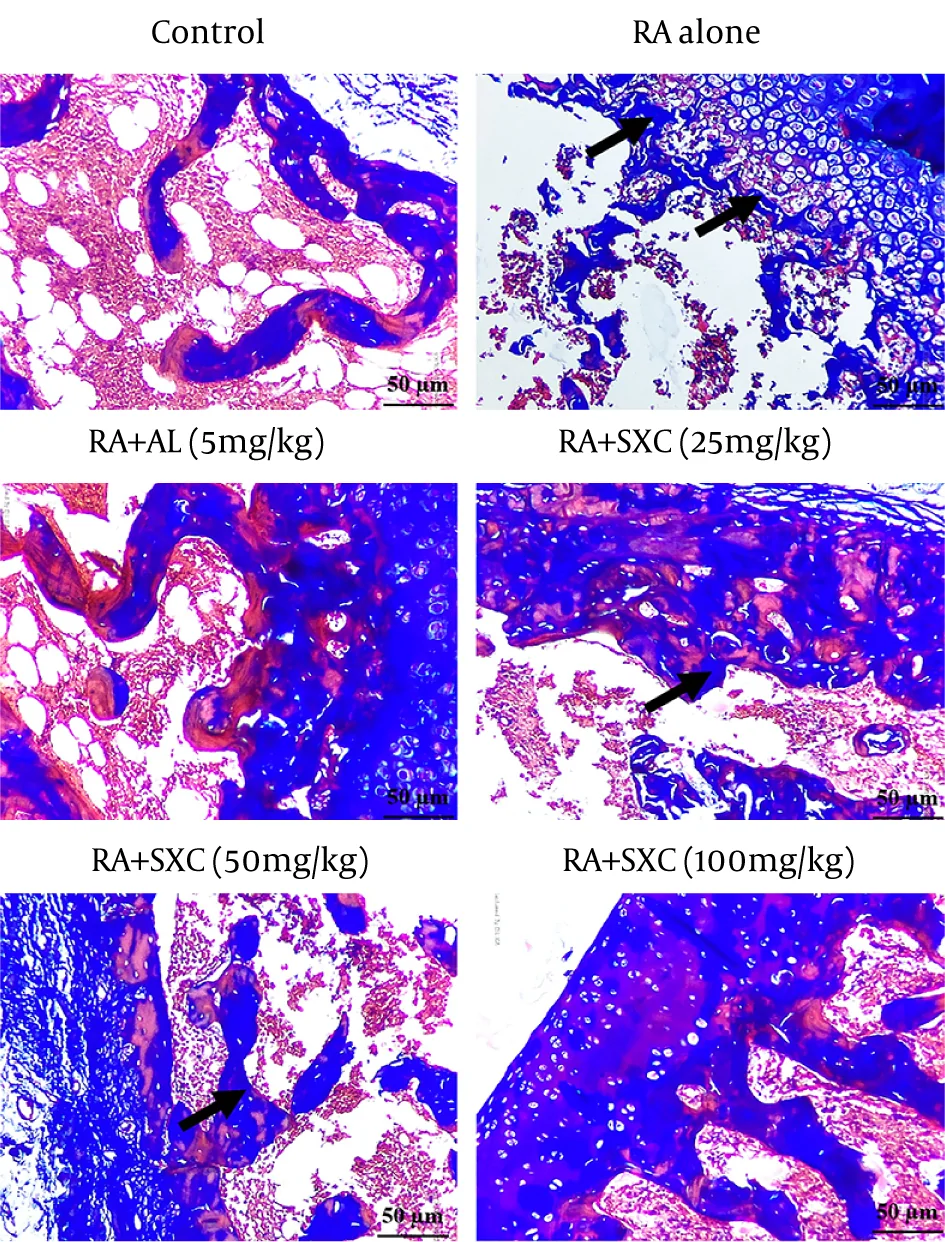
Histopathological assessment of collagen deposition in RA-induced bone injury. Representative photomicrographs of MT-stained sections of the right pelvic limb femurs (40x, scale bar = 50 μm). NC group: displays organized bone architecture with dense, mature collagen fibers (blue staining) and well-structured trabeculae. RA group: shows significant degradation of the collagenous matrix, characterized by fragmented trabeculae and reduced blue staining intensity. Black arrows indicate areas of pronounced collagen loss and bone-matrix erosion within the articular and subchondral regions. SXLE-treated groups: exhibit dose-dependent recovery of collagen content. Treatment with SXLE effectively attenuated RA-induced matrix degradation, as evidenced by more robust and continuous collagen-fiber staining (blue) and improved trabecular integrity.

### 4.9. Histopathological Evaluation of SXLE on RA-Induced Hepatic Injury

As shown in Figure 8, the NC group had normal liver architecture, with a distinct central vein and regularly arranged hepatocyte cords separated by intact sinusoids. In contrast, the RA group exhibited marked hepatic damage, including inflammatory cell infiltration around the portal area, sinusoidal congestion and dilation, and focal hepatocyte necrosis/vacuolation. Treatment with SXLE alleviated these pathological changes in a dose-dependent manner. The treated groups showed progressive reductions in inflammatory foci and sinusoidal dilation, together with restoration of the normal cord-like arrangement of hepatocytes, indicating a hepatoprotective effect of SXLE against RA-induced liver injury.

**Figure 8.**
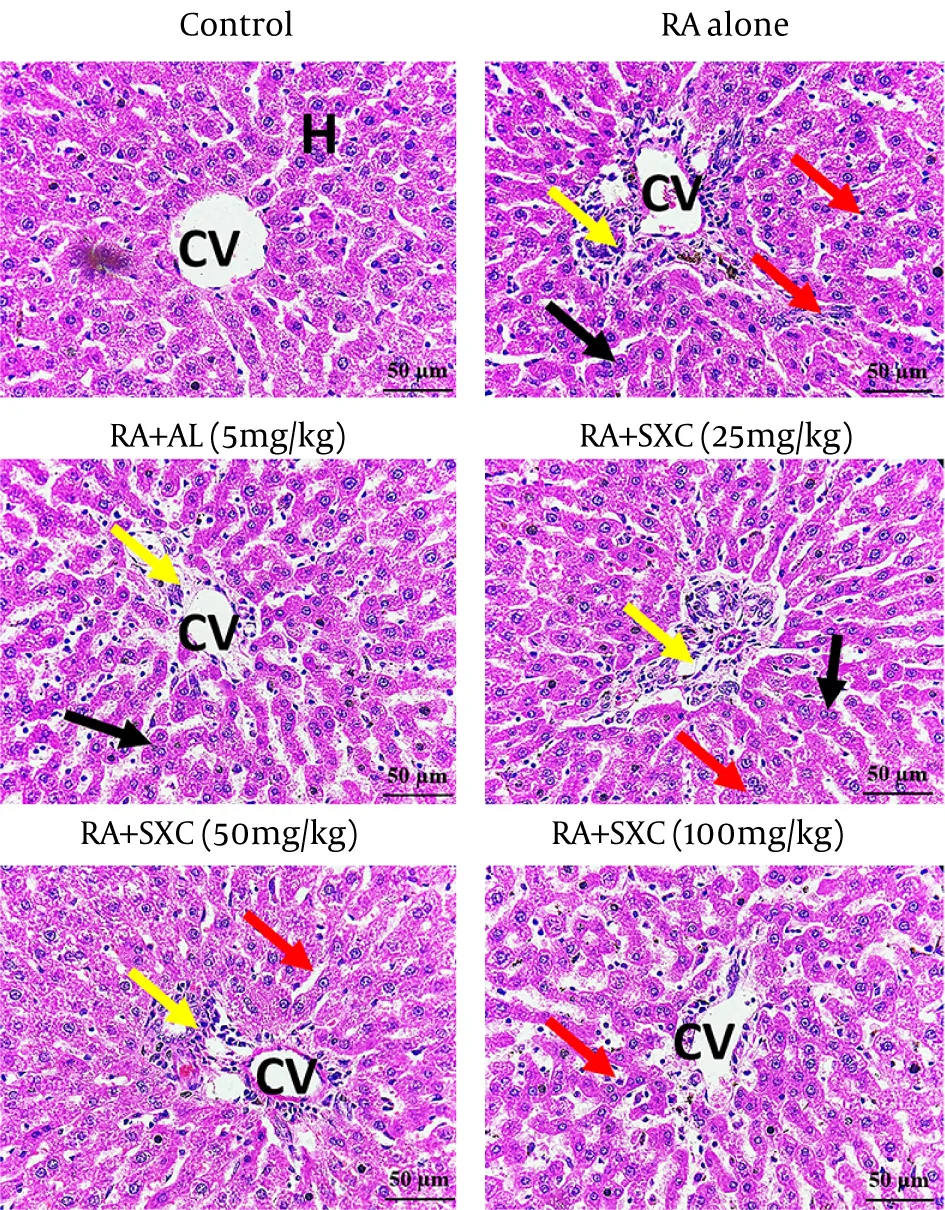
Histopathological evaluation of SXLE on RA-induced hepatic injury. Representative H&E-stained liver sections (40x, scale bar = 50 μm) illustrating the hepatoprotective effects of SXLE in RA-induced rats. NC group: shows normal hepatic architecture with a clear central vein (CV) and radiating cords of healthy hepatocytes (H) separated by sinusoids. RA group: exhibits significant liver damage characterized by inflammatory cell infiltration (yellow arrow) around the portal area, sinusoidal congestion/dilation (red arrow), and localized hepatocyte necrosis or vacuolation (black arrow). SXLE-treated groups: show a dose-dependent reduction in pathological features. There is a marked decrease in inflammatory foci (yellow arrow) and sinusoidal dilation (red arrow), with restoration of the cord-like arrangement of hepatocytes (black arrow), indicating the protective efficacy of SXLE against RA-induced systemic liver injury.

### 4.10. Histopathological Evaluation of SXLE on RA-Induced Renal Injury

Figure 9 shows normal renal architecture in the NC group, with intact glomeruli, clearly defined Bowman’s spaces, and well-preserved renal tubules. In contrast, the RA group exhibited severe renal injury, including glomerular hypercellularity and congestion, interstitial inflammatory cell infiltration, tubular degeneration/dilation, and distortion or reduction of Bowman’s space. SXLE treatment significantly attenuated these histological abnormalities in a dose-dependent manner. The treated groups showed progressive reductions in inflammatory infiltration, improvement in tubular integrity, and preservation of glomerular morphology, indicating a nephroprotective effect of SXLE against RA-associated renal damage.

**Figure 9.**
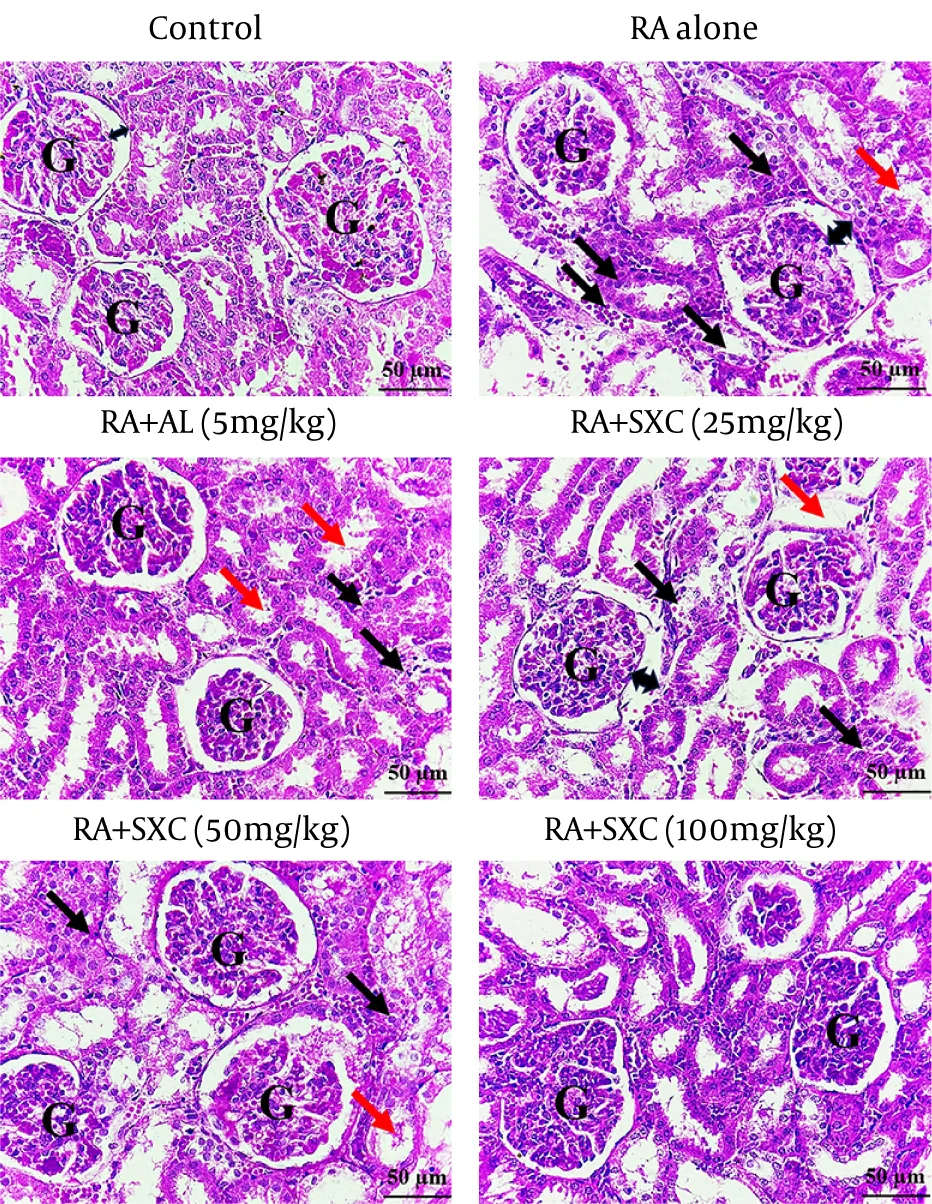
Histopathological evaluation of SXLE on RA-induced renal injury. Representative H&E-stained kidney sections (40x, scale bar = 50 μm) demonstrating the nephroprotective effects of SXLE in RA-induced rats. NC group: shows normal renal histology with intact glomeruli (G), well-defined Bowman’s spaces (double-headed arrows), and healthy renal tubules. RA group: exhibits prominent renal damage characterized by glomerular hypercellularity and congestion (G), interstitial inflammatory cell infiltration (black arrows), and tubular degeneration or dilation (red arrows). A noticeable reduction or distortion of Bowman’s space is also observed. SXLE-treated groups: show dose-dependent restoration of renal architecture. Treatment resulted in a reduction of inflammatory foci (black arrows), stabilization of tubular structures (red arrows), and preservation of glomerular morphology (G), indicating the protective effect of SXLE against RA-associated systemic renal complications.

### 4.11. Effects of SXLE on mRNA Expression of Pro-Inflammatory Mediators

RT-qPCR results showed that RA-treated rats had significantly increased (P < 0.05) mRNA expression of TNF-α, IL-6, and TP53 (tumor protein p53) compared with NC rats (Figure 10A-C). Treatment with SXLE at 25, 50, and 100 mg/kg body weight significantly reduced (P < 0.05) the expression of these inflammatory mediators in a concentration-dependent manner. AL (5 mg/kg body weight) and SXLE (100 mg/kg body weight) produced comparable mRNA expression of these mediators. These data indicate that SXLE exerts anti-inflammatory effects in RA-induced rats.

**Figure 10.**
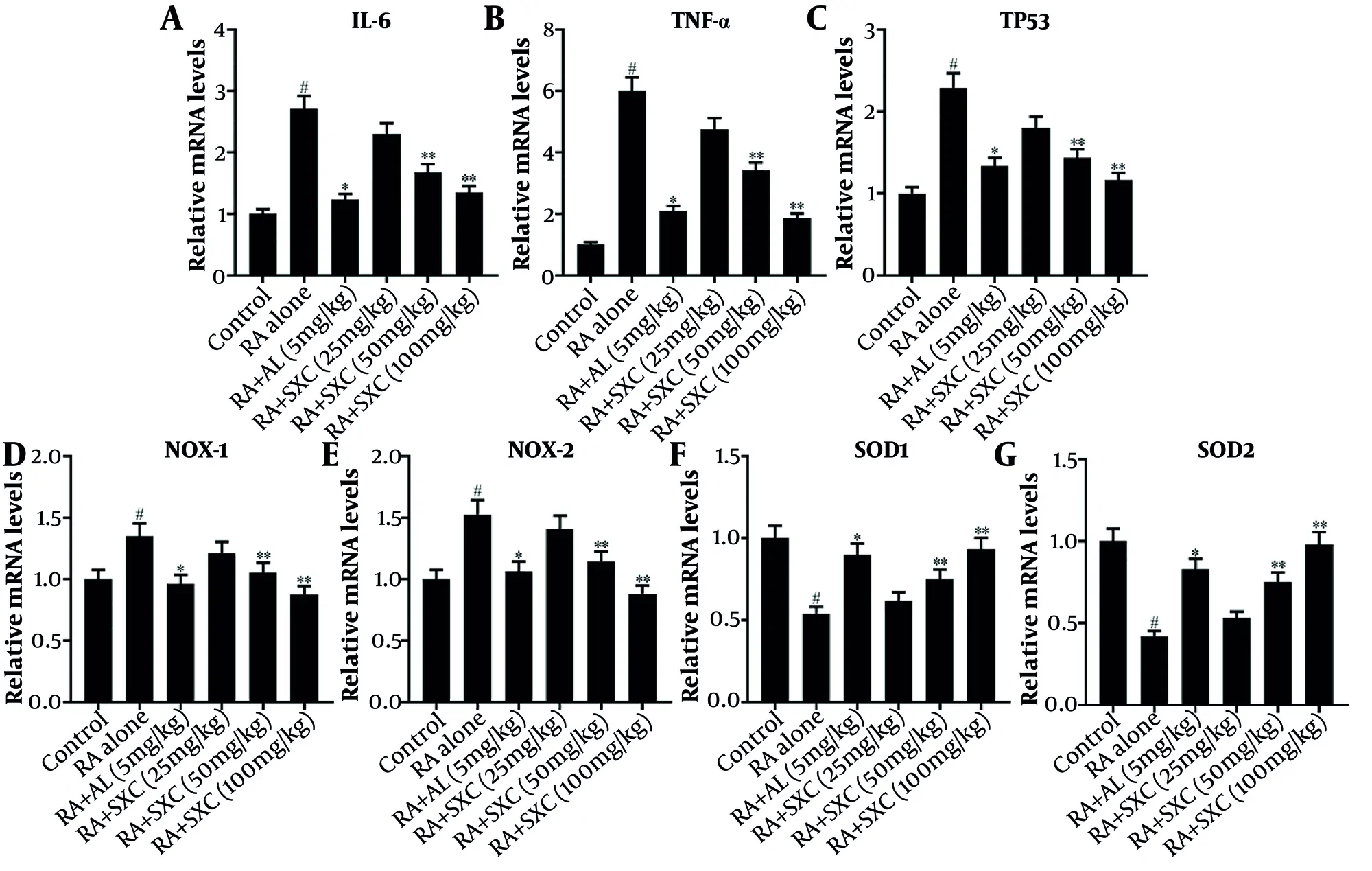
Influence of SXLE on mRNA expression of inflammatory mediators. Relative mRNA expression of A, IL-6; B, TNF-α; C, TP53; D, NOX-1; E, NOX-2; F, SOD1; and G, SOD2 was assessed by qRT-PCR analysis. Data are presented as mean ± SD of 6 observations, and statistical significance is indicated as #P < 0.05 vs NC, * P < 0.05 vs RA alone, and ** P < 0.01 vs RA alone.

### 4.12. Effects of SXLE on mRNA Expression of Oxidant and Antioxidant Enzymes

RT-qPCR results showed that RA-induced rats had significantly increased (P < 0.05) expression of the oxidant enzymes NOX1 and NOX2 and significantly decreased (P < 0.05) expression of the antioxidant enzymes SOD1 and SOD2 compared with NC rats (Figure 10D-G). SXLE treatment at 25, 50, and 100 mg/kg body weight significantly reduced (P < 0.05) NOX1 and NOX2 expression while increasing (P < 0.05) SOD1 and SOD2 expression in a concentration-dependent manner. AL (5 mg/kg body weight) and SXLE (100 mg/kg body weight) produced comparable expression profiles. These findings indicate that SXLE exerts antioxidant activity in RA-induced rats.

## 5. Discussion

OP is characterized by progressive reductions in bone mass and density and deterioration of trabecular microarchitecture, which increase fracture risk (1, 2). This disorder reflects an imbalance between osteoblast-mediated bone formation and osteoclast-mediated bone resorption (13-15). Although several clinically accepted therapies are available, long-term use of some antiresorptive agents has been associated with adverse effects, such as osteonecrosis of the jaw (8, 9). Consequently, there is continued interest in alternative agents with antioxidant and anti-inflammatory properties. Natural products and secondary plant metabolites, particularly phenolic compounds, have received increasing attention because of their diverse biological activities (16-20). Herbal phenolics have been reported to suppress osteoclastogenesis and reduce bone loss in experimental models (21-24). *Solanum xanthocarpum* contains bioactive constituents such as apigenin, solamargine, carpesterol, diosgenin, stigmasterol, lapel, and lupeol, which have been associated with bone-protective effects (25). Accordingly, the present study evaluated the protective effect of SXLE against RA-induced OP in rats.

Drug-induced OP is an emerging health concern because several medications adversely affect bone metabolism. Medication-associated oxidative stress plays an important role in many chronic disorders, including OP (34). Previous studies have shown that RA promotes OP by increasing oxidative stress, which is also associated with impaired antioxidant enzyme activity in animals and patients with OP (33, 34). RA promotes osteoclast differentiation and can therefore contribute to bone loss. The RA-induced OP model reproduces several features of human OP, including histomorphological changes and altered estrogenic responses (33, 34). In the present study, we evaluated whether SXLE could protect against RA-induced OP by modulating bone metabolism, oxidative stress, and inflammation. RA-treated rats showed lower body weight, serum Ca and P levels, femur/tibia length, BMD, estradiol, and osteocalcin, together with higher ALP, urinary bone minerals, and TRAP. SXLE treatment improved indices of bone formation and reduced indices of bone resorption, consistent with enhanced bone mineralization and metabolism. Our findings are comparable with those reported by Kahyaei-Aghdam et al. 2024 (40), who showed that hydroalcoholic *Lepidium draba* extract reduced oxidative stress and tissue injury in an experimental model. The protective effects observed in the present study may likewise be related to the antioxidant activity of phytoconstituents.

Nutritional factors also contribute to OP development. In the present study, serum calcium and phosphorus levels were reduced in RA-induced rats, whereas urinary calcium and phosphorus levels were significantly elevated compared with the NC group. SXLE treatment at 25, 50, and 100 mg/kg body weight increased serum calcium and phosphorus and reduced urinary calcium and phosphorus in a concentration-dependent manner. Previous studies have shown that bone resorption exceeds bone formation when estrogen levels decline (3, 4). Ca and P levels improved in SXLE-treated rats, particularly in the SXLE 100 mg/kg body weight and AL 5 mg/kg body weight groups. Femur, tibia, and liver coefficients also increased dose-dependently after SXLE treatment. Similarly, SXLE increased femur and tibia length, BMD, bone calcium content, cortical bone area ratio, relative bone density, and maximum load compared with the RA-alone group. These effects may be related to bioactive constituents of SX, including phenolics, flavonoids, alkaloids, and steroids (25-28). Histopathological examination showed sparse and disrupted bone in RA-treated rats, including reduced cortical bone thickness after 14 days of RA administration. These abnormalities improved in the SXLE 50 and 100 mg/kg body weight groups and in the AL 5 mg/kg body weight group, supporting a protective effect of SXLE against RA-induced OP.

Disruption of the balance between bone formation and resorption contributes to bone disease (12). OP is associated with changes in bone-turnover markers, including P, Ca, ALP, estradiol, TRAP, and OCN. In the present study, the elevated TRAP and ALP activities observed under osteoporotic conditions were reduced by SXLE. ALP is an osteoblastic biomarker involved in bone calcification (41-43), whereas TRAP is predominantly expressed by osteoclasts and reflects osteoclast activity (44). SXLE treatment was associated with recovery of calcium handling and bone calcification. Estradiol is also important for maintaining bone homeostasis, and reduced estradiol is a major contributor to osteoporosis in premenopausal and postmenopausal women. Our findings suggest that restoration of estradiol may contribute to the effect of SXLE on high bone turnover. OCN, which is secreted by osteoblasts, has an important role in bone regeneration (45). OCN was reduced in RA-treated rats but increased progressively with increasing SXLE dose, suggesting enhanced ossification and bone-matrix formation. Diosgenin has likewise been reported to increase osteoblast number or activity (8), and diets rich in olive oil have been associated with increased serum OCN (46). Thus, the SXLE-associated increase in OCN may contribute to suppression of bone loss.

Inflammation is closely associated with the development and progression of OP because inflammatory mediators can stimulate osteoclastogenesis and bone resorption. TNF-α and IL-6 are important mediators of immune and inflammatory responses and contribute to physiological and pathological bone resorption. IL-6 promotes osteoclast formation, particularly in estrogen-deficient states and severe skeletal disorders (11, 12). Estrogen deficiency may also increase IL-6, IL-1β, and TNF-α, further contributing to OP progression. TP53 is a transcription factor that can influence osteogenesis in bone marrow mesenchymal stem cells, and inflammatory signaling can increase TP53 expression and thereby affect bone metabolic balance (47). In the present study, SXLE at 25, 50, and 100 mg/kg body weight reduced the mRNA expression of TNF-α, IL-6, and TP53 in a concentration-dependent manner, supporting an anti-inflammatory effect.

The marked structural abnormalities in bone, liver, and kidney in the RA-treated group indicate systemic toxicity following retinoic acid exposure. The observed trabecular disruption, collagen-matrix loss, inflammatory infiltration, hepatocellular degeneration, and tubular/glomerular injury may reflect excessive retinoid signaling, oxidative stress, inflammatory activation, and disruption of normal cellular differentiation and extracellular-matrix homeostasis (48, 49). In bone, retinoid excess is known to impair skeletal remodeling by increasing osteoclast-mediated resorption while reducing osteoblast differentiation, matrix deposition, and mineralization. These mechanisms provide a plausible basis for the bone erosion, loss of collagenous matrix, and marrow architectural distortion observed in the RA group. However, retinoid effects on trabecular bone vary with species, dose, skeletal site, and exposure duration; some rodent studies identify trabecular loss, whereas others mainly report cortical bone loss.

Treatment with SXLE significantly improved histological architecture in a dose-dependent manner, suggesting protection against retinoic-acidinduced tissue injury. In the retinoid-toxicity literature, natural products and herbal formulations have been reported to attenuate multiorgan damage by suppressing inflammatory mediators, limiting oxidative stress, and preserving cellular and extracellular-matrix integrity.

In experimental models of retinoid toxicity, excess retinoic acid has been associated with hepatocellular degeneration, stellate-cell activation, fibrosis, skeletal remodelling disturbances, and renal tubular/glomerular injury, processes linked to oxidative stress, inflammatory signalling, and altered cellular differentiation. Natural products and herbal formulations have been reported to attenuate such multiorgan damage by suppressing pro-inflammatory mediators, enhancing antioxidant defences, and stabilizing tissue architecture.

The hepatoprotective effects observed in SXLE-treated groups are also consistent with reports that natural compounds can reduce inflammation and oxidative injury in liver tissue by modulating NF-κB-linked inflammatory signaling and enhancing antioxidant defenses. Likewise, the nephroprotective changes observed in kidney sections parallel reports that control of systemic rheumatoid arthritis activity can improve renal outcomes and that anti-inflammatory therapies may protect kidney structure and function. Together, these observations suggest that SXLE may exert multiorgan protective effects through combined anti-inflammatory, antioxidant, and tissue-stabilizing actions (50).

Overall, the present findings indicate that SXLE not only limited RA-induced bone destruction but also reduced hepatic and renal injury, suggesting a broader systemic effect. The dose-dependent improvement across tissues supports further investigation of SXLE as an adjunct candidate. As retained from the source manuscript, the discussion also compares these findings with rheumatoid arthritis studies describing bone erosion and secondary hepatic and renal injury through oxidative and inflammatory pathways. In agreement with reports on plant-derived therapeutics, SXLE produced dose-dependent improvements in tissue architecture, reduced inflammatory damage, and preserved bone matrix, collagen content, and organ integrity (50).

Oxidative stress impairs osteoblast differentiation and survival while promoting ROS-dependent osteoclast activity. NOX1 and NOX2 are important sources of ROS in RA-induced OP models. In the presence of RA, RANKL activates NOX1 and NOX2, increasing superoxide production and thereby promoting osteoclast activity while suppressing osteoblast activity (36). RA-induced OP is therefore closely associated with oxidative stress and altered SOD signaling. In the present study, the RA-alone group showed increased NOX1 and NOX2 expression and decreased SOD1 and SOD2 expression compared with the NC group. SXLE treatment reduced NOX1 and NOX2 while increasing SOD1 and SOD2 in a concentration-dependent manner. Similar changes have been reported in RA-treated RAW264.7 cells, in which melatonin reversed increased NOX1/NOX2 and reduced SOD1/SOD2 expression (36). These findings suggest that the antioxidant activity of SXLE contributes to improved bone quality and strength.

Downregulation of NOX1 and NOX2, together with upregulation of SOD1 and SOD2, provides a potential molecular basis for the bone-protective effects of SXLE. NADPH oxidases are major sources of ROS in bone tissue, and ROS promote osteoclast differentiation through NF-κB and nuclear factor of activated T-cells 1 (NFATc1) signaling. NOX1-mediated ROS production is particularly important for RANKL-induced osteoclast differentiation. In this study, SXLE reduced TRAP activity and the mRNA expression of inflammatory mediators TNF-α, IL-6, and TP53, changes that accompanied NOX1/NOX2 downregulation. By reducing NOX-derived ROS production, SXLE may suppress ROS-dependent NFATc1 activation and thereby reduce osteoclastogenesis.

Furthermore, NOX2-derived ROS have been implicated in bone loss through enhanced osteoclast formation in the bone-marrow microenvironment. ROS accumulation can activate RANKL signaling and upregulate osteoclast-specific genes, creating a positive feedback loop that favors bone resorption. The observed downregulation of NOX1/NOX2 by SXLE suggests that the extract may interrupt this ROS-RANKL-osteoclast axis, providing a mechanistic explanation for the reductions in cortical bone resorption and TRAP activity.

Conversely, oxidative stress impairs osteoblast function and accelerates osteoblast apoptosis through mitochondrial dysfunction and DNA damage. Superoxide dismutase enzymes (SOD1, cytosolic; SOD2, mitochondrial) convert superoxide radicals (O_2_-) to hydrogen peroxide (H_2_O_2_), thereby protecting osteoblasts from ROS-induced damage and supporting their differentiation. SXLE significantly upregulated SOD1 and SOD2 expression, consistent with the observed increases in OCN, BMD, and trabecular integrity. By strengthening endogenous antioxidant defenses, SXLE may create a more favorable microenvironment for osteoblast survival, matrix mineralization, and bone formation. This dual action—reducing NOX-dependent ROS production while enhancing SOD-dependent ROS scavenging—may help interrupt the cycle of osteoclast activation and osteoblast suppression.

This study is the first to demonstrate the anti-osteoporotic effects of SXLE in a retinoic acid-induced osteoporosis model, extending previous observations beyond ovariectomy models. The findings suggest 2 complementary actions: stimulation of osteoblastic activity, reflected by increased OCN, BMD, and cortical bone area, and suppression of osteoclastic activity, reflected by reduced TRAP, ALP, and inflammatory mediators. The results also link SXLE with modulation of the NOX1/NOX2-SOD1/SOD2 oxidative-stress pathway. At 100 mg/kg body weight, SXLE showed efficacy comparable to alendronate in several measured outcomes (Figure 11).

**Figure 11.**
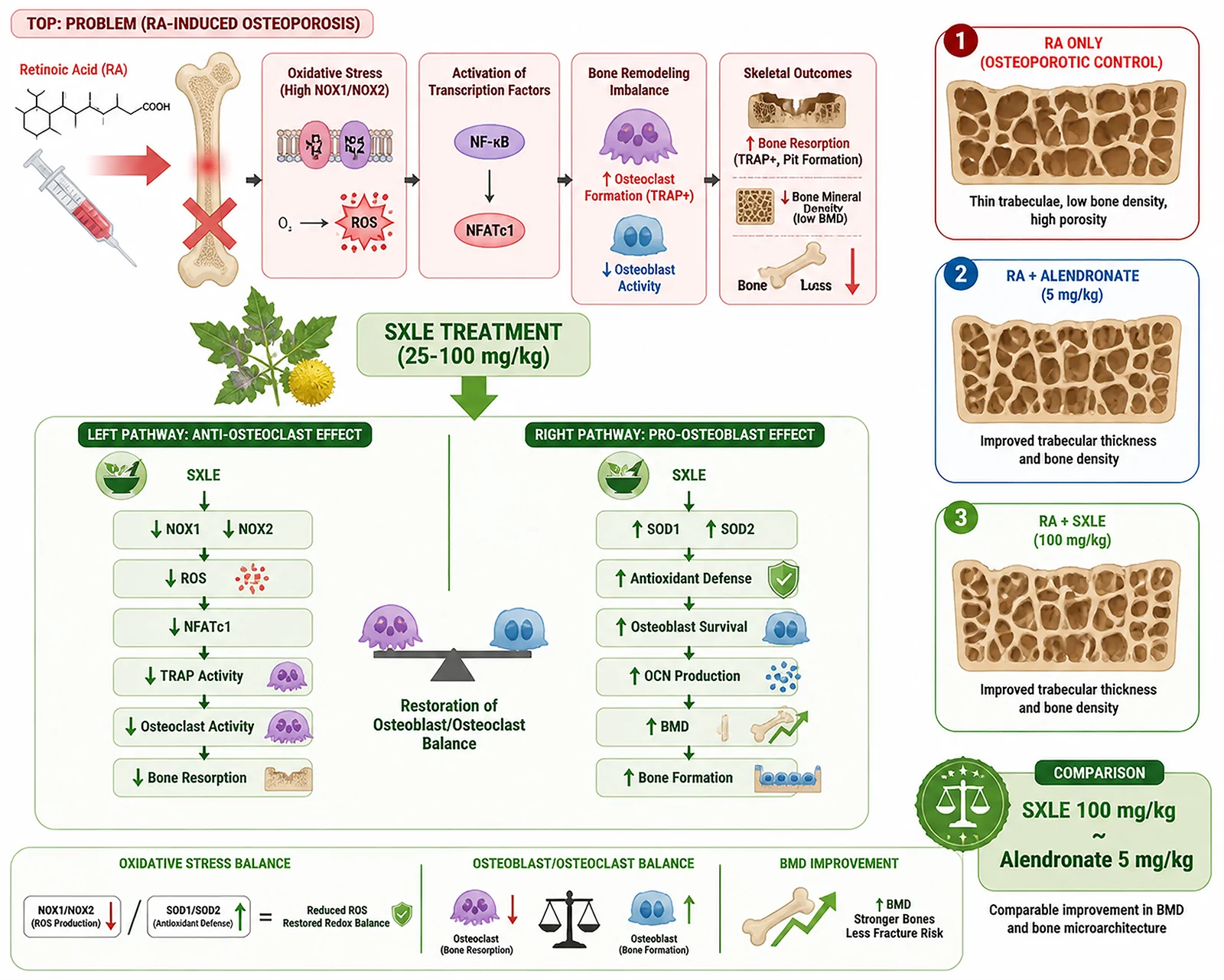
Mechanistic schematic of SXLE dual anti-osteoporotic action via NOX/SOD pathway modulation. RA-induced osteoporosis is driven by NOX1/NOX2-mediated ROS overproduction, which activates NFATc1 to promote osteoclastogenesis (↑TRAP) while inhibiting osteoblast function, resulting in bone loss. SXLE treatment (25, 50, and 100 mg/kg body weight) interrupts this pathway by: 1) downregulating NOX1/NOX2 → reducing ROS → suppressing NFATc1 → inhibiting osteoclast differentiation and bone resorption (left pathway); and 2) upregulating SOD1/SOD2 → enhancing antioxidant defense → protecting osteoblasts → increasing OCN production and bone formation (right pathway). RA + SXLE (100 mg/kg body weight) achieves bone microarchitectural improvement comparable to RA + AL (5 mg/kg body weight), demonstrating dual-action efficacy (osteoclast suppression + osteoblast stimulation) distinct from alendronate's mono-action (osteoclast inhibition only). This study provides the first report linking SXLE to NOX/SOD-mediated oxidative-stress pathway modulation in bone, establishing a novel molecular mechanism for its anti-osteoporotic activity.

The present findings are consistent with those of Kahyaei-Aghdam et al. 2024 (40), who reported antioxidant and anti-inflammatory effects of hydroalcoholic *Lepidium draba* extract in rats with isoproterenol-induced myocardial infarction. The extract improved hemodynamic parameters, reduced serum lactate dehydrogenase, and attenuated cardiac fibrosis, effects attributed in part to phenolic and flavonoid constituents and free-radical scavenging activity. Similarly, SXLE may protect tissues by restoring endogenous antioxidant defenses and reducing oxidative damage. The present findings are also consistent with those of Shirmohammadzadeh et al. 2025 (51), who reported modulation of COX-2-mediated inflammatory responses by chitosan-curcumin nanoparticles in a different disease model. Although the models differ, these studies support the broader concept that natural bioactive compounds can exert protective effects through regulation of oxidative stress and inflammation.

Our findings are also consistent with those reported by Naseri et al. 2025 (52), who found that *Pistacia atlantica* oleoresin nanoparticles protected against ethanol-induced gastric ulcers in rats, with the effects attributed to antioxidant and tissue-preserving properties. Despite differences in the disease models, both studies support the therapeutic potential of plant-derived bioactive compounds for limiting oxidative stress-mediated tissue injury.

### 5.1. Conclusions

In conclusion, SXLE increased body weight, femur and tibia length, and organ coefficients and improved histopathological alterations and bone-turnover markers in RA-induced rats. SXLE also modulated mineral profiles, antioxidant markers, and inflammatory mediators in a dose-dependent manner. The findings indicate enhanced osteoblastic activity and reduced osteoclastic activity in association with modulation of the NOX1/NOX2-SOD1/SOD2 signaling pathway. Although the RA model is not a typical estrogen-deficient model, SXLE significantly increased estrogenic levels, which may contribute to its bone-protective effects. The interaction between specific SXLE components and the endocrine system warrants further investigation. Additional studies using ovariectomy models, cellular experiments, compound isolation, detailed phytochemical fingerprinting, comprehensive chromatographic profiling, and phytochemical standardization are needed to strengthen the translational evidence for SXLE in osteoporosis management.

## Data Availability

No datasets were generated or analysed during the current study.
